# Ecotoxicological and Genotoxic Evaluation of Buenos Aires City (Argentina) Hospital Wastewater

**DOI:** 10.1155/2014/248461

**Published:** 2014-08-21

**Authors:** Anahí Magdaleno, Ángela Beatriz Juárez, Valeria Dragani, Magalí Elizabeth Saenz, Marta Paz, Juan Moretton

**Affiliations:** ^1^Cátedra de Higiene y Sanidad, Facultad de Farmacia y Bioquímica, Universidad de Buenos Aires, Junin 956, 4° Piso, C1113AAC Buenos Aires, Argentina; ^2^Departamento de Biodiversidad y Biología Experimental y Departamento de Química Biológica, Facultad de Ciencias Exactas y Naturales, Universidad de Buenos Aires, C1428EGA Buenos Aires, Argentina; ^3^Instituto de Biodiversidad y Biología Experimental y Aplicada (IBBEA-CONICET), C1428EGA Buenos Aires, Argentina

## Abstract

Hospital wastewater (HWW) constitutes a potential risk to the ecosystems and human health due to the presence of toxic and genotoxic chemical compounds. In the present work we investigated toxicity and genotoxicity of wastewaters from the public hospital of Buenos Aires (Argentina). The effluent from the sewage treatment plant (STP) serving around 10 million inhabitants was also evaluated. The study was carried out between April and September 2012. Toxicity and genotoxicity assessment was performed using the green algae* Pseudokirchneriella subcapitata* and the* Allium cepa* test, respectively. Toxicity assay showed that 55% of the samples were toxic to the algae (%I of growth between 23.9 and 54.8). The* A. cepa* test showed that 40% of the samples were genotoxic. The analysis of chromosome aberrations (CA) and micronucleus (MN) showed no significant differences between days and significant differences between months. The sample from the STP was not genotoxic to* A. cepa* but toxic to the algae (%I = 41%), showing that sewage treatment was not totally effective. This study highlights the need for environmental control programs and the establishment of advanced and effective effluent treatment plants in the hospitals, which are merely dumping the wastewaters in the municipal sewerage system.

## 1. Introduction

Hospitals wastewater (HWW) contains large amounts of hazardous chemical compounds, such as pharmaceuticals nonmetabolized by patients, disinfectants, active substances, pigments, dyes, reagents, and radioactive elements, due to laboratory and research activities or medicine excretion [1, 2]. According to Gupta et al. [3], hospitals consume a significant amount of water per day, ranging from 400 to 1,200 L day^−1^ bed^−1^, and generate equally significant amounts of wastewater. One of the main environmental problems caused by hospital effluents is due to their discharge in urban sewerage systems without preliminary treatment. In Buenos Aires (Argentina), these wastewaters enter directly into the municipal sewer system, which is further treated along with the domestic sewage in the sewage treatment plant (STP). The effluent from this plant is only primarily treated and then approximately 1.9 million m^3^ day^−1^ of wastewater is released into the Río de la Plata, the main source of drinking water for 10 million inhabitants. The absence of a hospital treatment process* in situ* increases the concentration of many not biodegradable compounds such as pharmaceuticals [4–6], released into the aquatic environment. However, Ort et al. [7] said that the pretreatment of HWW prior to discharge into the sewers does not provide a reasonable solution. The main reason is that this treatment should include full physical and biological treatment steps, not only advanced processes. Besides, capturing all sources within a hospital (wards and laboratories) may be further complicated by the fact that different facilities discharge through different pipes to the common sewer.

Analytical monitoring studies from Europe and US usually reported pharmaceutical concentrations in the ng L^−1^ to lower *μ*g L^−1^ range in river water as in drinking water [8]. However, no data are available about pharmaceutical concentrations in Río de la Plata. It is well established that pharmaceuticals and disinfectants are associated with deleterious effects in aquatic organisms [9–14]. Fluoroquinolones, for example, were classified as a priority risk group of antibiotics due to their potential genotoxic activity in bacteria and toxicity to algae and aquatic plants [15, 16]. It has been shown that the toxic mechanism of antibiotics to blue-green algae (cyanobacteria) may be via the interference of protein synthesis (e.g., chloramphenicol) and DNA replication (e.g., quinolones), but to green algae, the toxic effects are mostly attributed to the inhibition of the pathways involved in photosynthetic metabolism [17, 18]. Other pharmaceuticals such as citostatics that have been found in hospital wastewaters were reported as genotoxic to nontarget aquatic organisms [19]. Additionally, citostatics are suspected to be a possible cause of the increasing observed cancer cases in the last decades, constituting a potential risk to the environment and public health [20].

Due to the risk involved by drugs in the terrestrial and aquatic organisms, toxic and genotoxic biological tests are usually used as tools of monitoring and measuring of impact. Algae comprise an essential component of aquatic ecosystems, being the base of most aquatic food chains and playing crucial roles in nutrient cycling and oxygen production. Therefore, microalgae are often considered as a good indicator for anthropogenic pollution and water quality [21]. For example, some algae are more sensitive to pharmaceuticals than higher trophic organisms such as crustacean or fish [22, 23]. The standard algal bioassays measure the influence of contaminants on growth rate (cell division rate) or final cell biomass (cell yield) after 48 to 96 h exposure. Growth endpoints are the basis of most chronic algal toxicity tests and are particularly environmentally relevant because changes in population growth may influence species succession and community structure and function [24].

*Allium cepa* is acknowledged as an excellent genetic model to assess environmental pollutants [25, 26]. Moreover, this species also presents other advantages, including low raising costs, easy handling, and suitable chromosomal features; this plant bears large and few chromosomes (2*n* = 16) which facilitates the evaluation of chromosome damages and/or disturbances in cell division cycle, including eventual aneuploidy risks [27]. The observation of micronuclei (MN) and chromosome aberrations (CA), such as bridges, fragments, and vagrant, allows estimating the genotoxic effects of agents. The presence of MN in cells can result from acentric fragments (aneugenic agent) or whole chromosomes (clastogenic agent) that were not incorporated to the main nucleus during the cell cycle [28]. This test system has shown high sensitivity in detecting environmental chemicals [29]. However, only few researches have been carried out using* A. cepa* to evaluate genotoxicity on hospital wastewater samples [30, 31].

The aim of this work was to investigate the HWW toxicity and genotoxicity from San Martín Hospital (400 beds and 560 m^3^ wastewater daily), Buenos Aires city, Argentina. Additionally, a sample from the municipal STP of the city was analysed. The green algae* Pseudokirchneriella subcapitata* was selected for examining the toxicity of the samples. The genotoxicity was studied by the* Allium cepa* assay to detect chromosome damages and/or disturbances in cell division cycle. Until now, in Argentina, few characterizations of hospital effluents were carried out [32, 33].

## 2. Materials and Methods

### 2.1. Sampling of Hospital Wastewater

**Figure 1:**
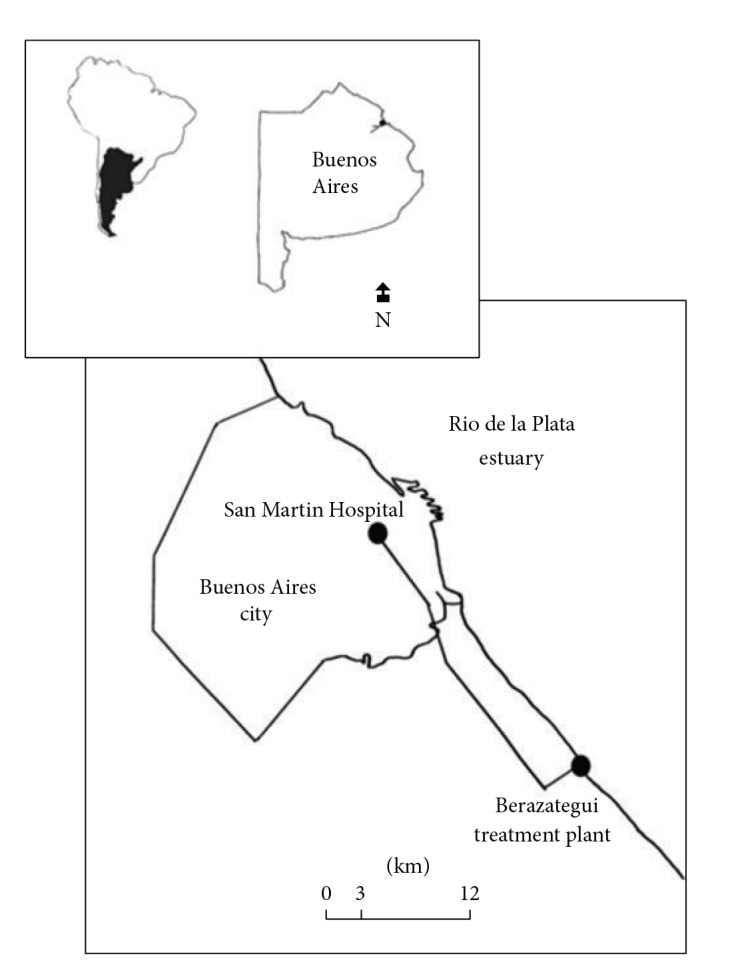
Location of San Martín Hospital in Buenos Aires city (Argentina), the municipal wastewaters system and Berazategui municipal treatment plant.

The San Martín Hospital is located in Buenos Aires city. The effluents are discharged directly into the municipal wastewaters system and conducted for 25 km towards the municipal STP located in Berazategui (Figure 1). This STP is a large plant situated in Río de la Plata, in Buenos Aires, Argentina. It receives all sewage from the Buenos Aires city (1.4 million inhabitants), including hospital and domestic sewage effluents, and it currently treats, on average, 1.9 million m^3^ day^−1^. This plant is based on the system of sewage treatment by dilution into a receiving water body. Therefore, this system comprises a pretreatment in which the solids (grit and grease) are retained, and then the pretreated waters are discharged into a receiving water body (Río de la Plata) by an outlet pipe. This system is based on the assumption of self-purification capacity of the river. Based on the data provided by the Water and Sanitation Company (AySA), the flow rate of STP effluent may vary between 29 and 33.5 m^3^ s^−1^.

A total of 20 hospital wastewater samples were taken during four months (April, June, July, and September 2012). The samples were collected in the HWW discharge site into the urban sewer system. The sampling was performed during the maximal hospital activity period (8:00 a.m.–6:00 p.m.), taking a sample every two hours (partial samples). The same volume (2 L) of each partial sample was mixed at the end of the day to obtain the composite sample submitted to biological test. As a preliminary evaluation of the sewage treatment efficacy, we introduce one effluent sample from the Berazategui STP in this study (Figure 1). This sample was collected at the beginning of the study (in April) in the effluent discharge site into the Río de la Plata. All the samples were taken in plastic bottles and stored on ice and darkness for transport back to the laboratory. Approximately 20 mL of each sample was filtered through a 0.22 *μ*m pore-size cellulose nitrate filter (Millipore) immediately after sampling and stored at −20°C during approximately a week before the assays.

The San Martín Hospital uses a significant quantity of antibiotics for the admitted patients [31, 32]. The estimation of the quantity of these pharmaceuticals during the study period (mean of each month and of the four studied months), the average daily water hospital consumption (560 m^3^ day^−1^), and the renal metabolization rate per patient allowed us to calculate a theoretical unmetabolized concentration of these compounds in the wastewater [13]. Then, we estimated the predicted environmental concentration for each antibiotic *i* (PEC (*a*)_*i*_) using the following equation:

(1) $$\begin{array}{c} {\text{PEC}\left(a\right)}_{i}=\frac{a_{i}\times f_{i}}{V}, \end{array}$$

where *a*_*i*_ is the antibiotic concentration consumed in the hospital per day, *f*_*i*_ is the fraction excreted in the urine, and *V* is the average volume of hospital wastewater consumption (560 m^3^ day^−1^) [13]. The *a*_*i*_ antibiotic amounts administrated to inpatients in San Martín Hospital were provided by the hospital pharmacy. Each *a*_*i*_ represents the mean concentration per day obtained for the four studied months (Table 1). We assumed *V* as the dilution factor.

The most frequently used disinfectants in the hospital were sodium hypochlorite, povidone-iodine, glutaraldehyde, nitrofurazone, and chlorhexidine. Taking into account the database of the hospital disinfectants quantities (Litter) consumed in the period of study, we estimated the predicted environmental concentration of each disinfectant *i* (PEC (*d*)_*i*_) using the following equation:

(2) $$\begin{array}{c} \text{PEC}{\left(d\right)}_{i}=\frac{d_{i}}{V}, \end{array}$$

where *d*_*i*_ is the disinfectant volume (L) consumed in the hospital per day and *V* is the average volume of hospital wastewater consumption (560 m^3^ day^−1^).

We estimated the theoretical total concentration of disinfectants and antibiotics in the influent of the STP taking into account the total number of hospitals (14) and beds for patients (4189) in Buenos Aires city, respectively. We only considered the contribution of the hospitals influent at the STP. Then, the predicted environmental concentrations for each compound *a*_*i*_ and *d*_*i*_ in the STP influent (PEC_(inf⁡)_) were calculated according to the following equation:

(3) $$\begin{array}{c} {\text{PEC}}_{\left(\inf\right)}=\frac{\text{PEC}\left(a_{i},d_{i}\right)}{D}, \end{array}$$

where PEC_(*a*_*i*_,*d*_*i*_)_ is the predicted environmental concentration for each antibiotic or disinfectant in the total HWW and the dilution factor *D* represents the total volume entering in the STP per day in average (1.9 million m^3^) (Table 1). As the treatment process is carried out only by the elimination of solids, the liquid volume in the influent (*D*) is the same to the effluent. Then (PEC_(inf⁡⁡)_) = (PEC_(eff)_).

### 2.2. Algal Growth Inhibition Test

The microalga* Pseudokirchneriella subcapitata* (Koršhikov) Hindak (previously named* Raphidocelis subcapitata* Koršhikov and* Selenastrum capricornutum* Printz) was the species selected to perform the bioassays. This species was obtained from the Culture Collection of Algae and Protozoa, UK (CCAP number 278/4), and maintained under axenic growth conditions in Bold's basal medium (BBM) [33]. The stock algae was cultivated in 125 mL Erlenmeyer flasks, containing 50 mL sterilized BBM medium, and agitated on a shaker at 80 rpm, under continuous cool-white fluorescent light (25 *μ*mol m^−2^ s^−1^). The flasks were maintained in the shaker incubator at 25 ± 2°C for 5 to 7 days to obtain the inoculum in the exponential growth phase (approximately 10^6^ cells mL^−1^). The bioassays were performed in sterile 96-well microplates according to Environmental Canada [34]. The filtered wastewater samples were previously enriched with the same nutrients comprising the BBM. Four replicate cultures were set up randomly for each sample and the control (BBM). For this, each well in the microplates was filled with 190 *μ*L of test water and 10 *μ*L of algal inoculum, so that the nominal initial cell concentration was 10^4^ cells mL^−1^. The microplates were incubated for 96 h in the shaker incubator under the same conditions performed to obtain the inoculum. Cell densities were estimated by absorbance at 620 nm. The percentages of growth inhibition with respect to the control were obtained at 96 h.

### 2.3. *Allium cepa* Test

The assay was carried out with* Allium cepa* seeds (variety Valcatorce), that are genetically and physiologically homogeneous. Both CA and MN tests using the meristematic cells were performed according to a modified version of Grant's protocol [35]. Onion seeds were germinated at room temperature (20 ± 5°C) in Petri dishes, each dish covered with filter paper and individually poured with a distinct water sample. Ultrapure water was used as a negative control and 2 × 10^−4 ^M of methyl methanesulfonate (MMS, CAS number 66-27-3) was used as a positive control. When the roots reached 1.5 cm in length (approximately 4 days after the beginning of the assay), they were collected and fixed in alcohol-acetic acid (3 : 1) for 24 h. Then, the fixed roots were stored in 70% ethyl alcohol. To prepare the slides, the meristematic regions were covered with coverslips and carefully squashed into a drop of 2% acetic orcein solution. The mitotic index (MI) was calculated by counting all stages of mitotic cells with respect to total cells. For the CA analyses, several aberrations such as chromosomal fragments, vagrant, and bridges in the anaphase and telophase were analyzed. All these categories were classified into just one category in order to evaluate the CA as a single endpoint, following the criteria used by Hoshina and Marin-Morales [36]. The MN induction was recorded by observing the interphase cells. The analyses were done by scoring 5000 cells per treatment, being 1000 cells per slide, comprising a total of 5 slides. CA and MN frequencies and mitotic index (MI) were calculated according to the formula: frequency = (*A*/*B*) × 100, where *A* is equivalent to the total number of cells with a parameter to be analyzed (CA, MN, and mitotic cells), and *B* corresponds to the entire number of analyzed cells.

### 2.4. Statistical Analysis

The results obtained in the* A. cepa* assay (MI, CA, and MN) were analyzed using a nonparametric statistical analysis, the Kruskal-Wallis test, with a significance level of 0.05. In order to evaluate significant differences between all the data obtained in different months and days, the Kruskal-Wallis test was used. Correlation analyses among the estimated concentrations of antibiotics and disinfectants and algal %I, AC, and MN were performed using Spearman's rank correlation coefficient, considering a *P* < 0.05. Correlation analyses among days and months of the algal %I, AC, and MN were also analysed. Graph Pad Prism 5 software was used for statistical analysis.

## 3. Results and Discussion

This is the first study in Argentina evaluating simultaneously the ecotoxicity and genotoxicity of HWW using two different assays. It is also the first to look at the efficacy of the treatment plant of municipal STP in reducing or not the toxic and mutagenic activity of the total sewage system from Buenos Aires city. We selected algae to evaluate the ecotoxicity of samples because these organisms represent the base of the trophic chain in aquatic ecosystems, and environmental impacts could lead to the inhibition or stimulation of algal growth [18]. On the other hand, we selected* A. cepa* bioassay, due to its sensitivity and effectiveness, to assess water and effluent pollution [37–39].

Among the different hospital wastewater constituents, antibiotics deserve special attention due to their biological activity, both to generate multiresistant bacteria and to induce adverse effects on living organisms [6, 40]. These drugs in particular have been identified as a category of trace chemical contaminants that warrant close scrutiny [41]. In this work we considered the antibiotics listed in Table 1 because previous work showed the presence of bacteria resistant to these antibiotics in the San Martín HWW [32]. The theoretical mean concentration of these compounds in the San Martín Hospital wastewater was 10.05 mg L^−1^ day^−1^. This value was approximately three times higher than that reported by Berto et al. [9] in Brazil (2.7 mg L^−1^). According to Orias and Perrodin [40], the predicted no-effect concentration (PNEC) of different antibiotics in aquatic organisms was as follows: ciprofloxacin (0.5 *μ*g L^−1^), cephalosporins group, including cefalotin, ceftazidime and ceftriaxone (36–101 *μ*g L^−1^), gentamycin (0.069 *μ*g L^−1^), vancomycin (372 *μ*g L^−1^), and ampicillin (0.00078 *μ*g L^−1^). Most of the theoretical antibiotic concentrations from the San Martín Hospital are more than 10-fold higher than their PNEC, while ciprofloxacin and ampicillin were 2- and 10,000-fold higher than the PNEC, respectively.

Sodium hypochlorite is often used for disinfecting hospital wastewater in order to prevent the spread of pathogenic microorganisms. However, this molecule reacts with organic matter giving halogenated organic compounds, which are toxic and genotoxic for aquatic organisms, and are considered as persistent environmental contaminants [42]. On the other hand, glutaraldehyde is the disinfectant used mainly for disinfecting instruments and medical probes. The glutaraldehyde concentrations detected in different countries from Europe are higher than the PNEC in aquatic organisms [40]. However, the concentration estimated in the San Martín Hospital effluent (0.0012 mg L^−1^, Table 1) was much lower than the lowest observed effect concentration for the green alga* P. subcapitata* (1.0 mg L^−1^) [43]. In the same way, the povidone-iodine estimated in the hospital wastewaters (Table 1) was much lower than the PNEC in aquatic organisms [40].

The* P. subcapitata* bioassays showed both inhibition and stimulation of growth at 96 h of culture. The obtained percentages of algal growth inhibition were between 23.9 and 54.8 (Table 2). A total of twenty samples were analysed, and eleven of them showed toxicity (55%), mainly in the samples corresponding to June and July. However, no significant differences (*P* < 0.05) between months and days were found. Many substances in effluent hospital could act to both inhibit and stimulate the algal growth. Total phosphorus, nitrogen, and organic matter present in high concentrations in hospital effluents [44] constitute algal nutrients and can act as growing factors. Therefore, these wastewater components stimulate the algal growth. Other components in the hospital effluent, such as pharmaceuticals and disinfectants, are toxic and can inhibit the algal growth. In the present work we estimated the HWW concentrations of the most used antibiotic and disinfectants in the San Martín Hospital. However, no significant correlations (*P* < 0.05) between these substances and %I of algal growth were observed. Among the antibiotics, ciprofloxacin is the most toxic antibiotic to green algae (EC_50_ = 2.79 mg L^−1^) [45] by inhibiting the photosynthetic apparatus [17]. However, the estimated concentration in the San Martín HWW was lower than this value (Table 1). On the other hand, amoxicillin is not toxic to* P. supcapitata* at higher concentrations than 1500 mg L^−1^ [46]. The hospital effluents are complex mixtures of many components, which could exert synergistic, antagonistic, and/or additive effects in the living organisms.

**Figure 2:**
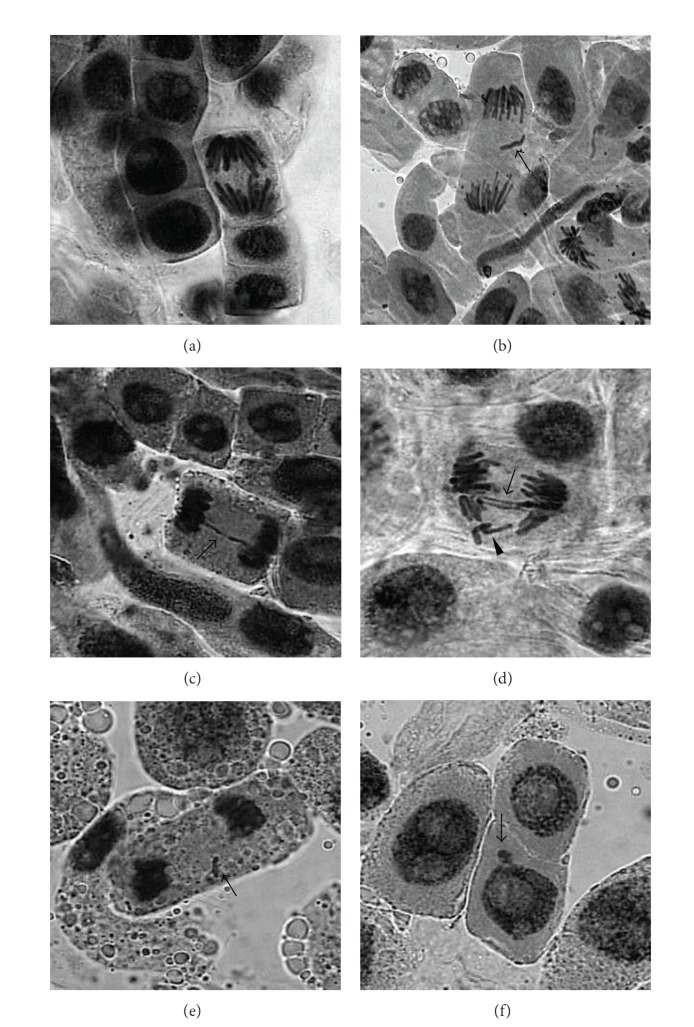
Main observations in* A. cepa* meristematic cells exposed to San Martín Hospital wastewaters: (a) normal anaphase; (b) anaphase with chromosome loss or vagrant (arrow); (c) anaphase with one chromosome bridge (arrow); (d) anaphase with two chromosome bridges (arrow) and losses (arrowhead); (e) telophase with chromosome fragment (arrow); (f) interphase with micronucleus (arrow).

The results from the MI, CA, and MN test in* A. cepa* root cells exposed to all the samples are shown in Table 3. The main CA observed in the meristematic cells of* A. cepa* exposed to the hospital effluent (bridges, vagrant, and fragments) and MN can be observed in Figure 2. No significant differences in MI values were observed among the negative control and the samples. All the samples collected in April showed higher CA frequencies than that observed in the negative control, although only the samples from Tuesday and Thursday were statistically significant (*P* < 0.05). Similarly, two samples collected in July and two samples collected in September showed significant differences with respect to the control (*P* < 0.05). In none of the June samples, genotoxic response was observed. For the MN test, the presence of this category in the negative control was not observed, but it was observed in eleven of twenty hospital effluent samples, the samples collected on Monday and Thursday of September being significantly different with respect to the control (*P* < 0.05).

Taking into account the analysis of frequencies of CA and MN, no significant differences were observed between days. However, significant differences have been found between April and June (*P* < 0.05) in the CA frequencies and April and September (*P* < 0.05) in the MN frequencies. The correlation analysis among the estimated concentrations of antibiotics, disinfectants, AC, and MN showed that only the frequencies of MN were positively correlated with ciprofloxacin (*R* = 0.7161, *P* = 0.0001). The highest mean values of MN were observed in July (0.66) and September (0.88), and also the highest ciprofloxacin mean concentrations (July = 0.437 mg L^−1^ and September = 0.443 mg L^−1^). Ciprofloxacin belongs to a fluoroquinolone group of antibiotics whose action mechanism is the inhibition of bacteria DNA replication by inhibiting the bacterial DNA-gyrase and topoisomerase II. However, similar effect could be produced on eukaryotic cells. Recently, it has been reported that fluoroquinolones are genotoxic in mammalian cells in culture [47]. Correlation between a particular compound and the observed genotoxicity is a difficult task. However, our results are related to other reported findings. Hartmann et al. [48, 49], for example, have found positive correlations between primary DNA damage and ciprofloxacin concentrations in German hospital wastewaters. On the other hand, Coutu et al. [50] intended to define the relative hazard for pharmaceutical substances in aquatic environment and potable water. These authors argue that physic-chemical characteristic of the substance is the most important criteria to estimate environmental hazard. According to their study, ciprofloxacin (between others) is one of the trace drugs that must be taken into account in drinking water for their possible effects on human health.

Eight of the total 20 hospital samples were genotoxic to* A. cepa* (40%), showing both high frequencies of CA and MN (Table 3). Among the CA, chromosomes fragment, bridges, and chromosomes vagrant were the main aberrations observed in anaphases and telophases of cell roots exposed to the San Martín Hospital samples (Figure 2). Few works were carried out using* A. cepa* to detect mutagenicity on wastewater samples from hospitals [30]. It is difficult to compare our results with those of other studies because of differences in the composition of the samples, the level of hospital activity, and medications used in treatments [51]. However, high percentage of positive samples has been reported by Bagatini et al. [30] in a hospital effluent from Brazil. The high frequencies of CA and MN observed in samples from San Martín Hospital clearly indicate the presence of toxic and mutagenic and/or genotoxic substances.

The wastewater sample from the Berazategui STP collected in April was toxic to the algae (%I = 41.73) (Table 2) but not genotoxic to* A. cepa* (Table 3). This is a preliminary test in which we aimed to analyze the effluent of Berazategui municipal STP. The high volume of liquid (1.9 million m^3^ day^−1^) entering in the plant reduces significantly the theoretical concentrations of the antibiotics and disinfectants considered in this work (Table 1). However, toxicity on algae was observed. The estimated concentrations of antibiotics and disinfectants in the STP effluent are not high enough to determine toxicity on algae. Therefore, toxicity could be due by other toxic compounds not considered in this work and/or synergistic processes among different components in the complex mixture of the wastewater sample.

The analysis of all components in a complex mixture, such as in wastewater sample, is a difficult task. One approach to this problem is the application of prediction models based on national yearly consumption (and/or sales) data, which can be used to calculate local PECs [11, 13]. However, those models do not consider many factors such as degradation processes (mainly photocatalytic and biological reactions) occurring in the environment. Coutu et al. [50] said that there are several factors causing inaccuracies in pharmaceuticals risk calculations, such as compounds with low excretion rates but with high conservation in the environment. A number of antibiotics such as ampicillin, erythromycin, sulphamethoxazole, and tetracycline are practically nonbiodegradable and have the potential to survive the sewage treatment [52]. Recently, Verlicchi et al. [41] showed that high differences between measured and predicted pharmaceutical concentrations may occur in municipal wastewater treatment plant and surface water, except for ciprofloxacin in wastewater and for azithromycin, trimethoprim, and carbamazepine in surface water. These authors argue that the possible reasons for those discrepancies are the uncertainties in the measured concentration values due to sampling mode and sensitivity of all the parameters required for predicting concentration due to dilution and excretion factor and removal efficiency. On the other hand, Ort et al. [7] showed that pharmaceuticals hospital data for inpatients appear to be good predictors for determining the fraction of pharmaceutical residues in HWW, and this approach can be used with some confidence for substances where no analytical data exists.

## 4. Conclusions

The San Martín HWW released into the municipal sewage system had toxic and genotoxic effects on* P. subcapitata* and* A. cepa*, respectively. The present study emphasizes the importance of analyzing the complex mixture of wastewaters from hospital using different biological tests. The results obtained by the* A. cepa* test showed that this assay could be suitable to detect genotoxicity in hospital effluents. The effluent from the municipal STP showed toxicity on* P. subcapitata*, indicating deficiencies in the treatment process. These preliminary results encourage us to continue studying the HWW and effluents from the municipal STP. This study highlights the need for environmental control programs and the establishment of advanced and effective effluent treatment plants in the hospitals, which are merely dumping the wastewaters in the municipal sewerage system.

## Figures and Tables

**Table 1:** Theoretical antibiotic and disinfectants concentrations (per day) in the hospital wastewater (HWW) and the sewage treatment plant (STP). The hospital consumption values represent the mean of the four months.

Compound	Hospital consumption (mg L^−1^)	Antibiotic fraction excreted by urine (%)	HWW (mg L^−1^)	STP (ng L^−1^)
Antibiotics				
Ampicillin	11,833	57	1.204	0.0066
Cefalotin	28,716	60	3.077	0.0169
Ceftazidime	641	85	0.097	0.0005
Ceftriaxone	30,650	61	3.339	0.0184
Ciprofloxacin	6,106	35	0.382	0.0021
Gentamycin	20	71	0.002	0.00001
Vancomycin	24,291	45	1.952	0.0107

	(%v/v)		(%v/v)	(%v/v)

Disinfectants				
Nitrofurazone	0.0002		0.0002	<0.00001
Povidone-iodine	0.0018		0.0018	<0.00001
Chlorhexidine	0.0001		0.0001	<0.00001
Glutaraldehyde	0.0012		0.0012	<0.00001
Sodium hypochlorite	0.0193		0.0193	<0.00001

**Table 2:** Percentages of algal growth inhibition (%I) measured in the raw filtered wastewater samples.

Sample	April	June	July	September
Monday	NA	28.1	27.3	33.1
Tuesday	NA	n.a.	29.9	25.9
Wednesday	44.5	35.6	54.8	NA
Thursday	NA	37.9	29.4	NA
Friday	NA	23.9	NA	NA
Berazategui plant	41.7	—	—	—

NA: not applicable; a stimulating effect was observed.

**Table 3:** Mitotic index (MI) and frequency of chromosomal aberrations (CA) and micronucleus (MN) in 5000 cells analyzed (mean ± deviation) of *Allium cepa* meristematic cells after exposure to the wastewater samples from hospital and Berazategui plant.

Samples	MI	CA	MN
April			
Negative control	13.06 ± 3.59	0.75 ± 0.96	0
Monday	9.30 ± 0.61	2.33 ± 1.15	0
Tuesday	9.91 ± 2.70	4.67 ± 1.53^a^	0
Wednesday	8.65 ± 2.90	2.00 ± 1.00	0
Thursday	11.05 ± 1.25	2.00 ± 0.05^a^	0
Friday	14.09 ± 3.30	1.25 ± 1.26	0
June			
Negative control	58.24 ± 10.24	0.85 ± 1.05	0
Monday	62.44 ± 1.52	0.67 ± 1.15	0.67 ± 0.58
Tuesday	62.86 ± 4.35	0.75 ± 0.50	0.50 ± 1.00
Wednesday	73.16 ± 4.55	0.50 ± 0.71	0
Thursday	65.11 ± 2.86	1.00 ± 1.22	0
Friday	57.36 ± 10.63	1.00 ± 0.82	0
July			
Negative control	58.24 ± 10.24	0.85 ± 1.05	0
Monday	47.25 ± 11.31	0.75 ± 0.95	0.75 ± 0.95
Tuesday	56.91 ± 8.42	2.00 ± 0.10^a^	0
Wednesday	59.92 ± 9.82	2.00 ± 1.73	1.33 ± 1.53
Thursday	61.16 ± 2.94	1.75 ± 0.96^a^	1.00 ± 1.41
Friday	53.74 ± 14.96	1.75 ± 1.71	0.25 ± 0.50
September			
Negative control	63.33 ± 1.16	0.92 ± 0.06	0
Monday	59.27 ± 5.87	2.25 ± 2.21	0.75 ± 0.50^a^
Tuesday	62.34 ± 6.94	1.40 ± 1.14	0.20 ± 0.45
Wednesday	62.49 ± 6.19	2.50 ± 1.29^a^	1.00 ± 2.00
Thursday	63.27 ± 7.41	1.33 ± 0.58	2.00 ± 1.00^a^
Friday	63.03 ± 7.97	3.00 ± 1.73^a^	0.33 ± 0.57
Berazategui plant			
Negative control	56.10 ± 11.39	0.75 ± 0.96	0
April	57.09 ± 9.76	0.43 ± 0.53	1.00 ± 1.15
MMS	69.03 ± 4.86	3.83 ± 2.14^a^	1.80 ± 0.84^a^

^a^Significantly different from negative control (*P* < 0.05), according to Kruskal-Wallis test.

## References

[1] Kümmerer K (2001). Drugs in the environment: emission of drugs, diagnostic aids and disinfectants into wastewater by hospitals in relation to other sources—a review. *Chemosphere*.

[2] Verlicchi P, Galletti A, Petrovic M, Barceló D (2010). Hospital effluents as a source of emerging pollutants: an overview of micropollutants and sustainable treatment options. *Journal of Hydrology*.

[3] Gupta P, Mathur N, Bhatnagar P, Nagar P, Srivastava S (2009). Genotoxicity evaluation of hospital wastewaters. *Ecotoxicology and Environmental Safety*.

[4] Carballa M, Omil F, Lema JM (2004). Behavior of pharmaceuticals, cosmetics and hormones in a sewage treatment plant. *Water Research*.

[5] Jorgensen SE, Halling-Sorensen B (2000). Drugs in the environment. *Chemosphere*.

[6] Kümmerer K (2004). Resistance in the environment. *Journal of Antimicrobial Chemotherapy*.

[7] Ort C, Lawrence MG, Reungoat J, Eaglesham G, Carter S, Keller J (2010). Determining the fraction of pharmaceutical residues in wastewater originating from a hospital. *Water Research*.

[8] Watkinson AJ, Murby EJ, Kolpin DW, Costanzo SD (2009). The occurrence of antibiotics in an urban watershed: from wastewater to drinking water. *Science of the Total Environment*.

[9] Berto J, Rochenbach GC, Barreiros MAB, Corrêa AXR, Peluso-Silva S, Radetski CM (2009). Physico-chemical, microbiological and ecotoxicological evaluation of a septic tank/Fenton reaction combination for the treatment of hospital wastewaters. *Ecotoxicology and Environmental Safety*.

[10] Blaise C, Gagné F, Eullaffroy P, Férard JF (2006). Ecotoxicity of selected pharmaceuticals of urban origin discharged to the Saint-Lawrence River (Québec, Canada): a review. *Brazilian Journal of Aquatic Science and Technology*.

[11] Emmanuel E, Perrodin Y, Keck G, Blanchard J-M, Vermande P (2005). Ecotoxicological risk assessment of hospital wastewater: a proposed framework for raw effluents discharging into urban sewer network. *Journal of Hazardous Materials*.

[12] Enick OV, Moore MM (2007). Assessing the assessments: pharmaceuticals in the environment. *Environmental Impact Assessment Review*.

[13] Escher BI, Baumgartner R, Koller M, Treyer K, Lienert J, McArdell CS (2011). Environmental toxicology and risk assessment of pharmaceuticals from hospital wastewater. *Water Research*.

[14] Ferrari B, Mons R, Vollat B (2004). Environmental risk assessment of six human pharmaceuticals: are the current environmental risk assessment procedures sufficient for the protection of the aquatic environment?. *Environmental Toxicology and Chemistry*.

[15] Ebert I, Bachmann J, Kühnen U (2011). Toxicity of the fluoroquinolone antibiotics enrofloxacin and ciprofloxacin to photoautotrophic aquatic organisms. *Environmental Toxicology and Chemistry*.

[16] Li M, Wei D, Zhao H, Du Y (2014). Genotoxic of quinolones: substituens contribution and transformation products QSAR evaluation using 2D and 3D models. *Chemosphere*.

[17] Halling-Sørensen B (2000). Algal toxicity of antibacterial agents used in intensive farming. *Chemosphere*.

[18] Liu BY, Nie XP, Liu WQ, Snoeijs P, Guan C, Tsui MTK (2011). Toxic effects of erythromycin, ciprofloxacin and sulfamethoxazole on photosynthetic apparatus in Selenastrum capricornutum. *Ecotoxicology and Environmental Safety*.

[19] Zounková R, Odráška P, Doležalová L, Hilscherová K, Maršálk B, Bláha L (2007). Ecotoxicity and genotoxicity assessment of cytostatic pharmaceuticals. *Environmental Toxicology and Chemistry*.

[20] Jolibois B, Guerbet M (2006). Hospital wastewater genotoxicity. *Annals of Occupational Hygiene*.

[21] Ma J, Lu N, Qin W, Xu R, Wang Y, Chen X (2006). Differential responses of eight cyanobacterial and green algal species, to carbamate insecticides. *Ecotoxicology and Environmental Safety*.

[22] Ferreira CSG, Nunes BA, Henriques-Almeida JMDM, Guilhermino L (2007). Acute toxicity of oxytetracycline and florfenicol to the microalgae *Tetraselmis chuii* and to the crustacean *Artemia parthenogenetica*. *Ecotoxicology and Environmental Safety*.

[23] Lanzky PF, Halling-Sørensen B (1997). The toxic effect of the antibiotic metronidazole on aquatic organisms. *Chemosphere*.

[24] Franklin NM, Stauber JL, Apte SC, Lim RP (2002). Effect of initial cell density on the bioavailability and toxicity of copper in microalgal bioassays. *Environmental Toxicology and Chemistry*.

[25] Grant WF (1994). The present status of higher plant bioassays for the detection of environmental mutagens. *Mutation Research: Fundamental and Molecular Mechanisms of Mutagenesis*.

[26] Grant WF (1999). Higher plant assays for the detection of chromosomal aberrations and gene mutations-a brief historical background on their use for screening and monitoring environmental chemicals. *Mutation Research*.

[27] Fiskesjǒ G (1985). The Allium test as a standard in environmental monitoring. *Hereditas*.

[28] Rank J, Nielsen MH (1997). *Allium cepa* anaphase-telophase root tip chromosome aberration assay on *N*-methyl-*N*-nitrosourea, maleic hydrazide, sodium azide, and ethyl methanesulfonate. *Mutation Research/Genetic Toxicology and Environmental Mutagenesis*.

[29] Leme DM, Marin-Morales MA (2009). *Allium cepa* test in environmental monitoring: a review on its application. *Mutation Research*.

[30] Bagatini MD, Vasconcelos TG, Laughinghouse HD, Martins AF, Tedesco SB (2009). Biomonitoring hospital effluents by the *Allium cepa L*. test. *Bulletin of Environmental Contamination and Toxicology*.

[31] Paz M, Muzio H, Mendelson A (2006). Evaluation of genotoxicity and toxicity of Buenos Aires city hospital wastewater samples. *Journal of the Brazilian Society of Ecotoxicology*.

[32] Moretton J, Nuñez L (2007). Desinfectant-resistant bacteria in Buenos Aires city hospital. *Brazilian Journal of Microbiology*.

[33] Archibald PA, Bold HC (1970). Phycological studies. XI. The Genus Chlorococcum Meneghini. *University of Texas Publication*.

[34] Environmental Canada (2007). *Biological Test Method: Growth Inhibition Test Using a Freshwater Algae*.

[35] Grant WF (1982). Chromosome aberration assays in *Allium*: A report of the US environmental protection agency gene-tox program. *Mutation Research*.

[36] Hoshina MM, Marin-Morales MA (2009). Micronucleus and chromosome aberrations induced in onion (*Allium cepa*) by a petroleum refinery effluent and by river water that receives this effluent. *Ecotoxicology and Environmental Safety*.

[37] Caritá R, Marin-Morales MA (2008). Induction of chromosome aberrations in the *Allium cepa* test system caused by the exposure of seeds to industrial effluents contaminated with azo dyes. *Chemosphere*.

[38] Leme DM, Angelis DF, Marin-Morales MA (2008). Action mechanisms of petroleum hydrocarbons present in waters impacted by an oil spill on the genetic material of *Allium cepa* root cells. *Aquatic Toxicology*.

[39] Rank J, Nielsen MH (1994). Evaluation of the Allium anaphase-telophase test in relation to genotoxicity screening of industrial wastewater. *Mutation Research—Environmental Mutagenesis and Related Subjects Including Methodology*.

[40] Orias F, Perrodin Y (2013). Characterisation of the ecotoxicity of hospital effluents: a review. *Science of the Total Environment*.

[41] Verlicchi P, Al Aukidy M, Jelic A, Petrović M, Barceló D (2014). Comparison of measured and predicted concentrations of selected pharmaceuticals in wastewater and surface water: a case study of a catchment area in the Po Valley (Italy). *Science of the Total Environment*.

[42] Emmanuel E, Keck G, Blanchard J-M, Vermande P, Perrodin Y (2004). Toxicological effects of disinfections using sodium hypochlorite on aquatic organisms and its contribution to AOX formation in hospital wastewater. *Environment International*.

[43] Sano LL, Krueger AM, Landrum PF (2005). Chronic toxicity of glutaraldehyde: differential sensitivity of three freshwater organisms. *Aquatic Toxicology*.

[44] Gautam AJ, Kumar S, Sabumon PC (2007). Preliminary study of physico-chemical treatment options for hospital wastewater. *Journal of Environmental Management*.

[45] Holten Lützhøft HC, Halling-Sørensen B, Jørgensen SE (1999). Algal toxicity of antibacterial agents applied in Danish fish farming. *Archival of Environmental Contamination and Toxicology*.

[46] González-Pleiter M, Gonzalo S, Rodea-Palomares I (2013). Toxicity of five antibiotics and their mixtures towards photosynthetic aquatic organisms: implications for environmental risk assessment. *Water Research*.

[47] Williams GM, Brunnemann KD, Smart DJ (2013). Relationship of cellular topoisomerase IIα inhibition to cytotoxicity and published genotoxicity of fluoroquinolone antibiotics in V79 cells. *Chemico-Biological Interactions*.

[48] Hartmann A, Alder AC, Koller T, Widmer RM (1998). Identification of fluoroquinolone antibiotics as the main source of umuC genotoxicity in native hospital wastewater. *Environmental Toxicology and Chemistry*.

[49] Hartmann A, Golet EM, Gartiser S, Alder AC, Koller T, Widmer RM (1999). Primary DNA damage but not mutagenicity correlates with ciprofloxacin concentrations in German hospital wastewaters. *Archives of Environmental Contamination and Toxicology*.

[50] Coutu S, Rossi L, Barry DA, Chèvre N (2012). Methodology to account for uncertainties and tradeoffs in pharmaceutical environmental hazard assessment. *Journal of Environmental Management*.

[51] Jolibois B, Guerbet M, Vassal S (2003). Detection of hospital wastewater genotoxicity with the SOS chromotest and ames fluctuation test. *Chemosphere*.

[52] Richardson ML, Bowron JM (1985). The fate of pharmaceutical chemicals in the aquatic environment. *Journal of Pharmacy and Pharmacology*.

